# The spectrum of comorbidities at the initial diagnosis of heart failure a case control study

**DOI:** 10.1038/s41598-022-06618-5

**Published:** 2022-02-17

**Authors:** Sven H. Loosen, Christoph Roderburg, Ole Curth, Julia Gaensbacher, Markus Joerdens, Tom Luedde, Marcel Konrad, Karel Kostev, Mark Luedde

**Affiliations:** 1 Clinic for Gastroenterology, Hepatology and Infectious Diseases, University Hospital Düsseldorf, Medical Faculty of Heinrich Heine University Düsseldorf, Moorenstraße 5, 40225 Düsseldorf, Germany; 2 Internal Medicine I, Central Hospital Bremerhaven, Bremerhaven, Germany; 3 Internal Medicine III, University Hospital of Schleswig Holstein, Campus Kiel, Kiel, Germany; 4 Health & Social, FOM University of Applied Sciences for Economics and Management, Frankfurt am Main, Germany; 5 Epidemiology, IQVIA, Frankfurt, Germany; 6 KGP Bremerhaven, Postbrookstr. 105, 27574 Bremerhaven, Germany; 7 Christian-Albrechts University of Kiel, Kiel, Germany

**Keywords:** Cardiology, Diagnosis

## Abstract

The prognosis of heart failure (HF) patients is determined to a decisive extent by comorbidities. The present study investigates the association between a broad spectrum of diseases and the occurrence of HF in a large collective of outpatients. This retrospective case control study assessed the prevalence of 37 cardiac and extracardiac diseases in patients with an initial diagnosis of heart failure (ICD-10: I50) in 1,274 general practices in Germany between January 2005 and December 2019. The study is based on the Disease Analyzer database (IQVIA), which contains drug prescriptions, diagnoses, and basic medical and demographic data. Patients with and without heart failure were matched by sex, age, and index year. Hazard regression models were conducted to evaluate the association between different disease entities and heart failure. The present study included 162,246 patients with heart failure and 162,246 patients without heart failure. Mean age [SD] was 73.7 [12.1] years; 52.6% were women. Out of 37 predefined diagnoses, 36 were more prevalent in HF patients. The highest prevalence was primary hypertension (63.4% in HF patients vs. 53.3% in controls, *p* < 0.001) followed by lipid metabolism disorders (34.6% in HF patients vs. 29.1% in HF patients *p* < 0.001) and diabetes mellitus type II (32.2% in HF patients vs. 25.2% in controls, *p* < 0.001). In the regression analysis, 19 diseases were significantly associated with heart failure. Non-cardiovascular diagnoses strongly associated with HF were obesity (HR = 1.46), chronic bronchitis and COPD (HR = 1.41), gout (HR: 1.41), and chronic kidney disease (HR = 1.27). In the present study, we identified a variety of cardiac and extracardiac diseases associated with heart failure. Our data underscore the immense importance of comorbidities, even as early as at the stage of initial diagnosis of heart failure.

## Introduction

Heart Failure has recently been defined as a clinical syndrome with symptoms and/or signs caused by a structural and/or functional cardiac abnormality and corroborated by elevated natriuretic peptide levels and/or objective evidence of pulmonary or systemic congestion^1,2^. Although important innovative therapeutic approaches have been established in recent years, the prognosis of heart failure is still poor^3–6^. In this context, comorbidities or concomitant diseases of heart failure patients play a decisive role. Concomitant diseases and comorbidities are diagnostically definable clinical pictures that are present in addition to an underlying disease or index disease^7,8^. In the case of heart failure, these can significantly aggravate the prognosis of the underlying disease, but also and above all significantly worsen the subjective feeling of illness^9^. Heart failure is usually diagnosed at older age, which may partly explain the high frequency of comorbidities. A third of patients report that other medical conditions are primarily responsible for their clinical condition. While heart failure with reduced ejection fraction (HFrEF) is often associated with comorbidities, heart failure with preserved ejection fraction (HFpEF) appears to be mainly defined by these conditions^10^. In this context, intracardiac and extracardiac can be differentiated^9^. Some intracardiac comorbidities such as coronary artery disease are clearly identified as precipitating or risk factors for heart failure^11^. Extracardiac comorbidities such as arterial hypertension are also known risk factors for the development of heart failure^11^. Other conditions such as renal failure are so frequently associated with the prognosis of heart failure that common pathomechanisms are discussed^12^. In this large case–control study, we assess the prevalence of intra- and extracardiac diseases that were diagnosed within 12 months prior to the diagnosis of heart failure in comparison to patients without heart failure.

### Objective

Our objective is to gain a comprehensive profile of comorbidities commonly present at the initial diagnosis of heart failure.

## Methods

### Database

This study was based on data from the Disease Analyzer database (IQVIA), which contains drug prescriptions, diagnoses, and basic medical and demographic data obtained directly and in anonymous format from computer systems used in the practices of general practitioners and specialists^13^. The database covers approximately 3% of all outpatient practices in Germany. Diagnoses (according to International Classification of Diseases, 10th revision [ICD-10]), prescriptions (according to Anatomical Therapeutic Chemical [ATC] Classification system), and the quality of reported data are being monitored by IQVIA. In Germany, the sampling methods used to select physicians’ practices are appropriate for obtaining a representative database of general and specialized practices. It has previously been shown that the panel of practices included in the Disease Analyzer database is representative of general and specialized practices in Germany^14^. For example, Rathmann et al. were able to show a good agreement of the incidence or prevalence of cancer diagnoses between the outpatient DA database with German reference data^14^. Finally, this database has already been used in previous studies focusing on cardiovascular disorders^15–17^.

### Study population

**Figure 1:**
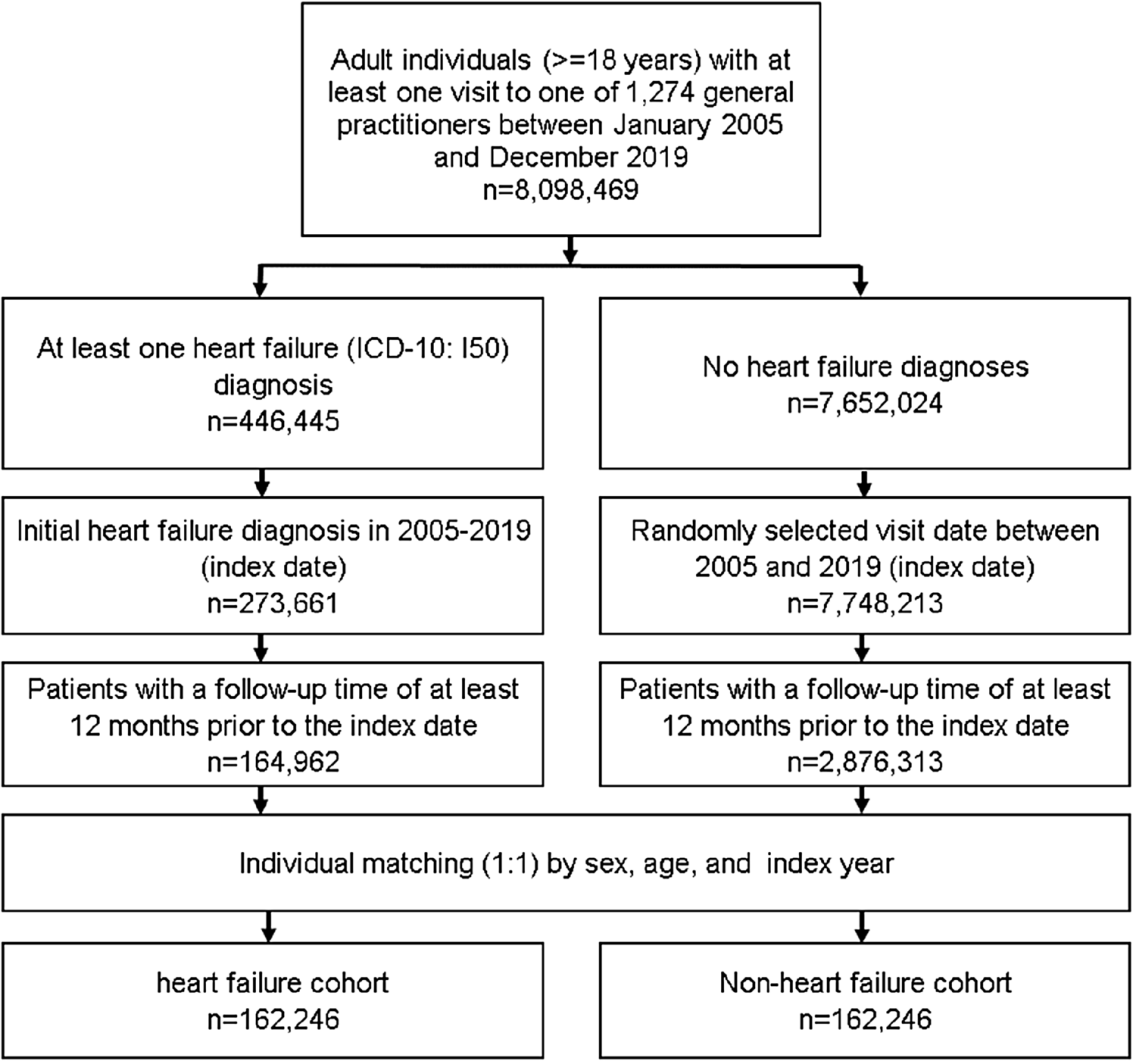
Selection of study patients.

This retrospective cohort study included adult patients (≥ 18 years) with an incident diagnosis of heart failure (ICD-10: I50) in 1,274 general practices in Germany between January 2005 and December 2019 (index date; Fig. 1). Another inclusion criterion was an observation time of at least 12 months prior to the index date.

Patients with and without heart failure were matched by sex, age, and index year, For the controls, the index date was that of a randomly selected visit between January 2005 and December 2019 (Fig. 1).

### Study outcomes and covariates

Diagnoses documented within 12 months prior to the index date were analyzed if they were documented in at least three percent of the study population. A total of 37 diagnoses were available for analysis, including cancer (ICD-10: C00-C98), iron deficiency anemia (ICD-10: D50), hypothyroidism (ICD-10: E03), hyperthyroidism (ICD-10: E05), diabetes mellitus type 2 (ICD-10: E11), obesity (ICD-10: E66), lipid metabolism disorders (ICD-10: E78), dementia (ICD-10: F00-F03, G30), depression (ICD-10: F32, F33), anxiety disorder (ICD-10: F41), sleep disorders (ICD-10: G47), hearing loss (ICD-10: H91, H92), primary hypertension (ICD-10: I10), hypertensive heart disease (ICD-10: I11), angina pectoris (ICD-10: I20), myocardial infarction (ICD-10: I21-I23), coronary heart disease (ICD-10: I24, I25), nonrheumatic aortic valve disorders (ICD-10: I35), atrial fibrillation and flutter (ICD-10: I48), ischemic stroke (ICD-10: I63, I64), atherosclerosis (ICD-10: I70, peripheral vascular diseases (ICD-10: I73), venous embolism and thrombosis (ICD-10: I80-I82), chronic bronchitis and COPD (ICD-10: J42-J44), asthma (ICD-10: J45), gastro-esophageal reflux disease (ICD-10: K21), gastritis and duodenitis (ICD-10: K29), diverticular disease (ICD-10: K57), hemorrhoids (ICD-10: K64), NALFD (ICD-10: K75.8, K76.0), cholelithiasis (ICD-10: K80), gout (M10), osteoarthritis (M15-M19), spondylopathies (ICD-10: M45-M49), osteoporosis (ICD-10: M80, M81), chronic kidney disease (ICD-10: N18, N19), benign prostatic hyperplasia in men (ICD-10: N40).

### Statistical analyses

Differences in the sample characteristics and diagnosis prevalence between those with and those without heart failure were tested using chi-squared tests for categorical variables and Wilcoxon tests for age. Multivariable logistic regression models were conducted to study the association between the pre-defined diagnoses and heart failure. This model contained 37 diagnoses which were adjusted for each other. A Bonferroni correction for p-value was performed, and a p-value of < 0.001 (calculated as < 0.05/37) was considered statistically significant. Analyses were performed separately for women and men. Analyses were carried out using SAS version 9.4 (SAS Institute, Cary, USA).

### Ethical standards

Only aggregated, anonymized patient data were used in these analyses. This study was performed in accordance with the Declaration of Helsinki and the guidelines for Good Practice of Secondary Data Analysis^18^. Since only anonymized data were used which could not be traced back to individual persons, the research protocol did not have to be approved by the local ethics committee, and it was not necessary to obtain informed consent from individual patients to participate in the study. This was confirmed by the local ethics committee of the Christian-Albrechts-University (CAU) of Kiel, Kiel, Germany (File reference D413/21).

### Patient and public involvement statement

Patients and the public were not involved in the generation of this research paper.

## Results

### Basic characteristics of the study sample

The present study included 162,246 patients with heart failure and 162,246 patients without heart failure. The basic characteristics of study patients are displayed in Table 1. Median age [25%–75% quartiles] of the study population was 76 [67–82] years; 52.6% were women. Table 2 shows 37 diagnoses (excluding cancer sites) the proportions of which, with exception of dementia, were higher in HF patients than in controls. The proportions of cancer diagnoses were very similar in patients with and without heart failure. The highest prevalence was primary hypertension (63.4% in HF patients vs. 53.3% in controls < 0.001) followed by lipid metabolism disorders (34.6% in HF patients vs. 29.1% in HF patients *p* < 0.001) and diabetes mellitus type II (32.2% in HF patients vs. 25.2% in controls, *p* < 0.001). In men, no significant difference was observed between patients with and without heart failure for cancer and hemorrhoids.

**Table 1:** Age and sex structure of the study sample (after 1:1 matching by sex, age, and index year).

Variable	Proportion affected among patients with heart failure (%) N = 162,246	Proportion affected among patients without heart failure (%) N = 162,246	*p* value
Age (Median, 25% and 75% quartiles)	76 (67–82)	76 (67–82)	1.000
Age ≤ 60	14.6	14.6	1.000
Age 61–70	18.3	18.3	
Age 71–80	34.5	34.5	
Age > 80	32.6	32.6	
Women	52.6	52.6	1.000
Men	47.4	47.4	

Proportions of patients given in %, unless otherwise indicated. SD: standard deviation.

**Table 2:** Prevalence of different diagnoses documented within one year prior to the index date in patients with and without heart failure followed in general practices in Germany (multivariable logistic regression models).

Diagnosis	All patients			Women			Men		
	Proportion among patients with heart failure	Proportion among patients without heart failure	*p* value	Proportion among patients with heart failure	Proportion among patients without heart failure	*p* value	Proportion among patients with heart failure	Proportion among patients without heart failure	*p* value
Cancer (C00-C98)	19.5	19.0	< 0.001	18.1	17.1	< 0.001	21.2	21.1	0.668
Prostate	−	−	−	−	−	−	4.1	4.1	0.899
Lymphoid and haematopoietic tissue	2.0	2.0	0.911	1.8	1.7	0.439	2.3	2.3	0.369
Female genital organs	–	–	–	1.1	1.0	0.171	–	–	–
Breast	–	–	–	3.6	3.5	0.343	–	–	–
Skin	2.8	2.6	< 0.001	2.5	2.3	0.002	3.2	2.9	< 0.001
Respiratory organs	0.9	1.0	< 0.001	0.6	0.6	0.092	1.2	1.4	< 0.001
Digestive organs	2.3	2.5	< 0.001	2.0	2.1	0.011	2.6	3.0	< 0.001
Urinary tract	1.2	1.2	0.364	0.7	0.7	0.771	1.8	1.8	0.360
Iron deficiency anemia (D50)	5.9	4.4	< 0.001	6.7	5.0	< 0.001	4.9	3.7	< 0.001
Hypothyroidism (E03)	7.4	6.4	< 0.001	10.2	8.8	< 0.001	4.3	3.7	< 0.001
Hyperthyroidism (E05)	4.0	3.2	< 0.001	5.3	4.1	< 0.001	2.7	2.1	< 0.001
Diabetes mellitus type 2 (E11)	32.2	25.2	< 0.001	30.4	23.3	< 0.001	34.3	27.3	< 0.001
Obesity (E66)	12.0	7.1	< 0.001	11.9	7.2	< 0.001	12.0	7.0	< 0.001
Lipid metabolism disorders (E78)	34.6	29.1	< 0.001	33.0	28.4	< 0.001	36.4	29.9	< 0.001
Dementia (F00-F03, G30)	8.3	8.6	< 0.001	10.0	10.6	< 0.001	6.4	6.4	0.507
Depression (F32, F33)	18.1	16.4	< 0.001	22.7	20.3	< 0.001	13.0	12.0	< 0.001
Anxiety disorder (F41)	5.6	4.8	< 0.001	7.1	6.0	< 0.001	3.9	3.4	< 0.001
Sleep disorders (G47)	14.7	12.3	< 0.001	15.7	13.4	< 0.001	13.7	11.0	< 0.001
Hearing loss (H91, H92)	6.2	5.4	< 0.001	6.2	5.4	< 0.001	6.2	5.4	< 0.001
Primary hypertension (I10)	63.4	53.3	< 0.001	64.3	54.3	< 0.001	62.3	52.2	< 0.001
Hypertensive heart disease (I11)	8.0	3.3	< 0.001	8.1	3.3	< 0.001	7.9	3.3	< 0.001
Angina pectoris (I20)	6.1	3.1	< 0.001	5.6	2.9	< 0.001	6.8	3.4	< 0.001
Myocardial infarction (I21-I23)	8.0	4.1	< 0.001	5.2	2.6	< 0.001	11.2	5.7	< 0.001
Coronary heart disease (I24, I25)	28.8	16.9	< 0.001	23.5	13.6	< 0.001	34.6	20.6	< 0.001
Nonrheumatic aortic valve disorders (I35)	4.6	2.4	< 0.001	4.3	2.3	< 0.001	4.9	2.5	< 0.001
Atrial fibrillation and flutter (I48)	18.6	9.0	< 0.001	17.6	8.6	< 0.001	19.8	9.5	< 0.001
Ischemic stroke (I63, I64)	5.6	4.9	< 0.001	5.2	4.5	< 0.001	6.0	5.3	< 0.001
Atherosclerosis (I70)	6.5	4.6	< 0.001	5.8	4.2	< 0.001	7.3	5.1	< 0.001
Peripheral vascular diseases (I73)	7.7	5.2	< 0.001	6.0	4.1	< 0.001	9.6	6.5	< 0.001
Venous embolism and thrombosis (I80-I82)	5.8	4.5	< 0.001	6.6	5.2	< 0.001	4.8	3.8	< 0.001
Chronic bronchitis and COPD (J42-J44)	16.6	10.5	< 0.001	14.9	9.2	< 0.001	18.4	12.0	< 0.001
Asthma (J45)	7.6	5.8	< 0.001	8.4	6.2	< 0.001	6.7	5.3	< 0.001
Gastro-esophageal reflux disease (K21)	15.4	12.6	< 0.001	16.5	13.2	< 0.001	14.2	12.0	< 0.001
Gastritis and duodenitis (K29)	17.3	15.2	< 0.001	19.0	16.3	< 0.001	15.4	14.0	< 0.001
Diverticular disease (K57)	5.7	5.2	< 0.001	6.0	5.5	< 0.001	5.5	5.0	< 0.001
Hemorrhoids (K64)	5.2	5.0	0.002	4.9	4.6	< 0.001	5.6	5.5	0.400
NALFD (K75.8, K76.0)	5.4	3.8	< 0.001	4.8	3.4	< 0.001	6.0	4.3	< 0.001
Cholelithiasis (K80)	5.1	4.4	< 0.001	6.0	5.1	< 0.001	4.2	3.6	< 0.001
Gout (M10)	7.0	4.2	< 0.001	4.8	2.5	< 0.001	9.5	6.0	< 0.001
Osteoarthritis (M15-M19)	28.5	23.6	< 0.001	33.0	27.1	< 0.001	23.5	19.8	< 0.001
Spondylopathies (M45-M49)	13.9	11.5	< 0.001	15.1	12.4	< 0.001	12.7	10.4	< 0.001
Osteoporosis (M80, M81)	10.1	8.7	< 0.001	15.9	13.9	< 0.001	3.7	3.1	< 0.001
Chronic kidney disease (N18, N19)	13.1	8.3	< 0.001	12.1	7.3	< 0.001	13.1	8.3	< 0.001
Benign prostatic hyperplasia (N40) (in men)	–	–	–	–	–		16.9	15.1	< 0.001

### Association of predefined diagnoses with heart failure

**Figure 2:**
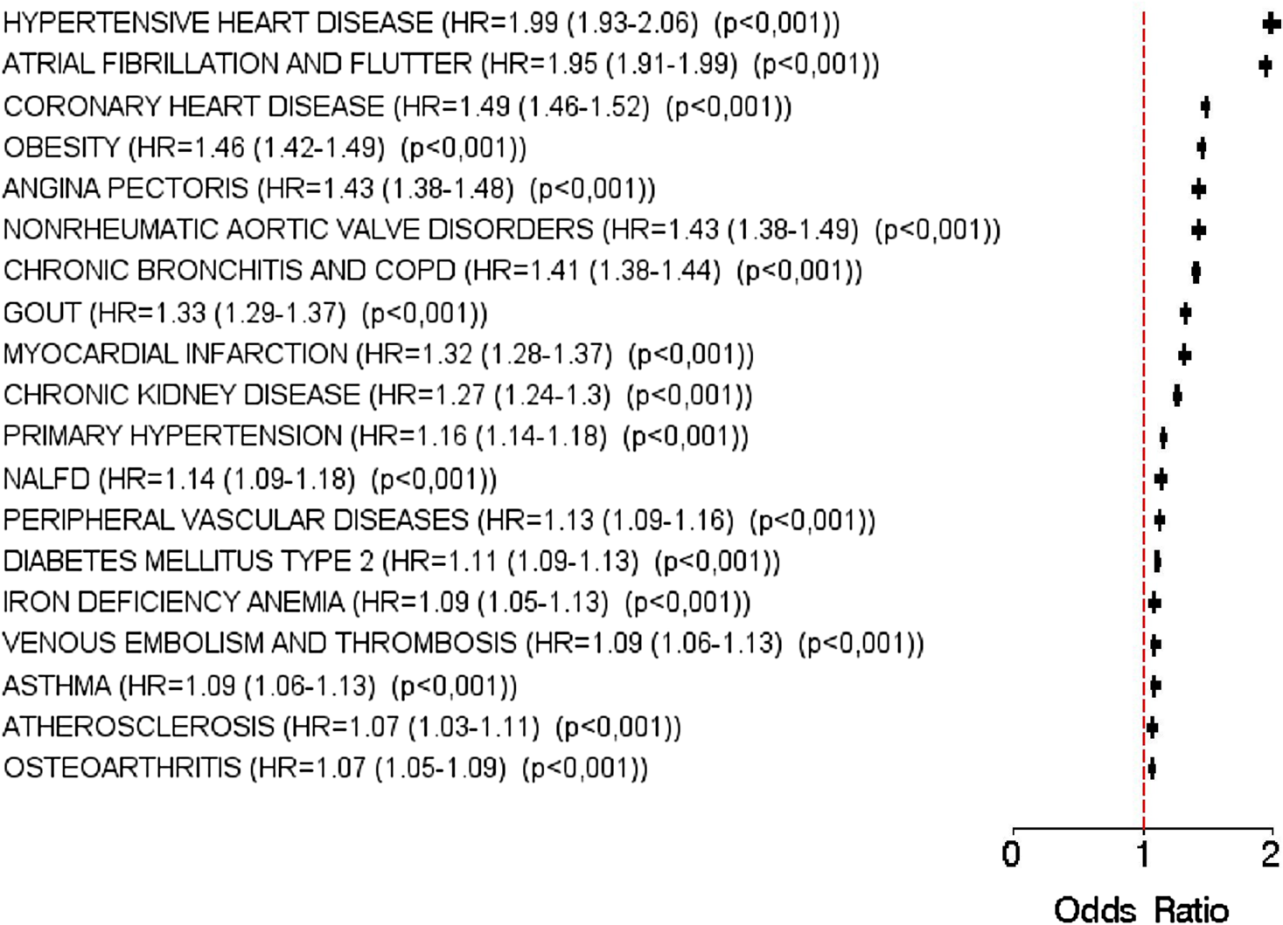
Association between different diagnoses and heart failure in patients followed in general practices in Germany (multivariable logistic regression models).

In our regression analyses, 19 diseases were significantly associated with heart failure (Fig. 2). The strongest association was observed for hypertensive heart disease (hazard ratio [HR] = 1.99), followed by atrial fibrillation and flutter (HR = 1.95), and coronary heart disease (HR = 1.49). Non-cardiovascular diagnoses strongly associated with HF were obesity (HR = 1.46), chronic bronchitis and COPD (HR = 1.41), gout (HR: 1.41), and chronic kidney disease (HR = 1.27). Furthermore, chronic kidney disease, NALFD, diabetes mellitus type 2, asthma, iron deficiency anemia, and osteoarthritis were also significantly associated with HF, but these associations were relatively weak (HR < 1.15).

Associations between diagnoses with the highest risks (OR > 1.20) and HF were similar in females and males (Table 3).

**Table 3:** Association between top 10 risk diagnoses and heart failure in female and male patients followed in general practices in Germany (multivariable logistic regression models).

Diagnosis	Women		Men	
	Odds ratio (95% CI)	*p* value	Odds ratio (95% CI)	*p* value
Hypertensive heart disease	2.01 (1.92–2.11)	< 0.001	1.98 (1.89–2.08)	< 0.001
Atrial fibrillation and flutter	1.93 (1.88–2.00)	< 0.001	1.98 (1.92–2.04)	< 0.001
Coronary heart disease	1.47 (1.43–1.51)	< 0.001	1.53 (1.49–1.57)	< 0.001
Obesity	1.42 (1.37–1.47)	< 0.001	1.50 (1.44–1.56)	< 0.001
Angina pectoris	1.40 (1.33–1.47)	< 0.001	1.46 (1.39–1.53)	< 0.001
Nonrheumatic aortic valve disorders	1.39 (1.31–1.47)	< 0.001	1.48 (1.40–1.57)	< 0.001
Chronic bronchitis and COPD	1.43 (1.39–1.48)	< 0.001	1.40 (1.36–1.45)	< 0.001
Gout	1.42 (1.34–1.50)	< 0.001	1.32 (1.27–1.37)	< 0.001
Myocardial infarction	1.28 (1.21–1.36)	< 0.001	1.35 (1.30–1.41)	< 0.001
Chronic kidney disease	1.29 (1.25–1.34)	< 0.001	1.25 (1.21–1.30)	< 0.001

## Discussion

Although heart failure is defined primarily by an elevation of the heart-specific marker (NT) pro BNP, even in the most recent definitions^1,2^, the importance of comorbidities in the development, course, and prognosis of heart failure has also been recognized in recent years^3^. In our analysis, we identified 19 diseases that were significantly associated with the incidence of heart failure. We consider only diagnoses that were present within 12 months prior to the initial diagnosis of heart failure, not those that were newly diagnosed during the course of heart failure. This explains, for example, why the proportion of patients with dementia was not higher in the HF group than in the control group. The risk of new-onset heart failure among patients with dementia does not appear to be increased. Conversely, it has been clearly demonstrated that the risk of new-onset dementia is increased during the course of heart failure^16,19^. Like heart failure, dementia is a huge problem today, with incidence and prevalence continuing to rise^20^. This may explain why the prevalence in our cohort is higher than that reported in the first EuroHeart Failure Survey^21^ from 2003, for example. Both are diseases found mainly in older patients. Common mechanisms of disease are proposed, e.g., frailty^22^. The coincidence of both diseases is certainly an issue that impacts the care of those affected as well as patient quality of life and prognosis^23^, meaning that special importance will have to be placed on this issue in the future, including in the area of scientific work^19^.

In our analysis, the strongest association with the incidence of heart failure was observed for hypertensive heart disease. The association between primary arterial hypertension and heart failure was also significant, but significantly less pronounced (HR 1.13). Especially in light of the considerable prevalence of arterial hypertension in our collective (63.4% in the cardiac efficiency group vs. 53.3% in the control group), our results highlight the importance of effectively breaking the pathological sequence of arterial hypertension-hypertensive heart disease-heart failure. The improvement of the medicinal adjustment of arterial hypertension and thus the prevention of end organ damage to the heart offers an excellent opportunity to protect many patients from developing heart failure^24,25^. Our data underline this very clearly.

The prevalence of obesity in our collectives is relatively low. The possibility of a recording bias cannot be ruled out here. Nevertheless, this extracardiac comorbidity was strongly associated with heart failure in our study. Interestingly, the respective hazard ratio was higher than that of diabetes. The two conditions of obesity and diabetes are considered so closely linked that they are also referred to as a parallel coepidemic of the western world, and the term diabesity has been coined in response^26^. Obesity may have deletive effects on myocardial remodelling similar to those of diabetes. Research into the stand-alone effects of obesity on the heart is still in its infancy; pericardial adipose tissue, for example, could play an important role as an endocrine organ. The strong association of obesity with heart failure in our study suggests the importance of further research in this area. This is an important key to the prevention of heart failure, given the enormous and increasing prevalence of obesity in industrialised countries^26^. Obesity and diabetes in particular have been linked to HFpEF, which is a disease diagnosed mainly in older individuals^10^. The median age in our study (and control) group was 73 years, which is lower than that in other Western European datasets^27^. This may be due in part to the fact that we studied outpatients with an initial diagnosis of heart failure and many other studies enrol patients as the disease progresses or those admitted to hospital for heart failure^27^. The possibility that the proportion of HFpEF patients is lower in our heart failure cohort because of the slightly younger age of our cohort and the lower proportion of obese patients cannot be excluded. Due to missing information in our database, which is based on ICD-10 codes, we were unable to determine the ejection fraction in our HF patients (see Limitations part of the Discussion section).

The association between COPD and new-onset heart failure is strong, with COPD being very prevalent in our study population (16.6% in the HF group, 10.5 in the control group), which also reflects the high prevalence in the general population. Clinically, this association is particularly problematic as both conditions can present with similar symptoms, which can significantly delay the diagnosis of heart failure^28^. In addition, certain drugs such as beta sympathomimetics can have significant side effects in heart failure patients, and overall, the large group of patients with COPD and heart failure should be considered particularly at risk^29^.

Another strong association was shown in our study between gout and the occurrence of heart failure. It must be emphasised again that our study describes statistical associations and does not prove causal relationships. A clear causal relationship between these diseases has not yet been described; however, gout has been associated with coronary heart disease and cardiovascular mortality in general^30^. Recent hypotheses on the genesis of heart failure place gout in a series with other chronic inflammatory conditions such as rheumatoid arthritis and chronic inflammatory bowel diseases^31^. It is well established that heart failure is also a state of chronic inflammation, and it has been postulated that there may be a cardioinflammatory subtype of heart failure that is particularly associated with chronic inflammation. This would potentially provide intriguing treatment options for some heart failure patients^31^. However, the fact that gout was significantly more strongly associated with heart failure than rheumatoid arthritis in our study may suggest that gout-specific pathomechanisms also contribute to the occurrence of heart failure beyond general inflammatory conditions.

No significant differences were found between the heart failure and control groups with regard to cancer in general and specific cancers. Cardio-oncology is an emerging field in the area of cardiology. Initially considered to include the risk of recurrence of heart failure in oncology patients treated with cardiotoxic therapies, increasingly heart failure is also being inversely linked to cancer. Recent data suggest that heart failure is also a prooncogenic entity, with an increased rate of cancer in heart failure patients. Low-grade inflammation and neurohumoral activation are cited as mechanisms linking the two conditions. In addition, there are, of course, common risk factors, such as nicotine. Our study does not show an increased cancer rate at the onset of heart failure. Other studies which focus more on the long-term course of the disease, are likely to show this association.

Due to the large size of our collective, the analysis of possible differences between the sexes with regard to comorbidities/risk factors is of possible interest and warranted. Recent work has shown that there are important differences between women and men in clinical characteristics, comorbidities, and response to therapy^32,33^. In both study groups, we found significant differences between men and women in the prevalence of dementia, anxiety disorders, and depression, with an overall higher prevalence in women both with and without heart failure. Clear differences in the respective gender-specific distribution between heart failure patients and control patients were not found. However, further studies such as ours will certainly be needed to identify gender differences that could also explain different responses to therapies^32^.

As a caveat, secondary data analyses such as ours are generally limited by a lack of completeness of the data on which they are based. For example, we cannot provide information on the individual disease stages of disease entities, or on the guideline-based therapy of the diseases and the course of the disease. As such, we are unable to differentiate between different subtypes of heart failure (HFrEF vs. HFpEF). Furthermore, no data were available in the database on the individual left ventricle ejection fraction. In addition, identification of patients was based on officially coded diagnoses only, most likely resulting in a recording bias. The possibility that diagnoses have been misclassified within the ICD-10 coding system cannot be excluded. However, we used data from a large database with over 162,000 patients for our case–control study. We are therefore confident that the associations shown are reliable and clinically meaningful.

In summary, we can give a very comprehensive and broad picture of associations of various internal diseases with the new onset of heart failure in a case–control study. Comorbidities are risk factors for the occurrence of heart failure as well as aggravating factors for the course of the disease. However, they could also offer new starting points for multimodal innovative therapy approaches for heart failure in the future.
